# Patients’ experiences of the caring encounter in health promotion practice: a qualitative study in Swedish primary health care

**DOI:** 10.1186/s12875-020-01296-6

**Published:** 2020-11-11

**Authors:** Kristina Lundberg, Mats Jong, Miek C. Jong, Lisbeth Porskrog Kristiansen

**Affiliations:** 1 Department of Caring Sciences, University of Gävle, Faculty of Health and Occupational Studies, Kungsbäcksvägen 47, SE-801 76 Gävle, Sweden; 2 Department of Health Sciences, Mid Sweden University, Holmgatan 10, SE-851 70 Sundsvall, Sweden; 3 National Research Center in Complementary and Alternative Medicine (NAFKAM), Department of Community Medicine, Faculty of Health Sciences, UiT The Arctic University of Norway, Hansine Hansens veg 19, 9019 Tromsø, Norway

**Keywords:** Caring encounter, Health promotion, Lifestyle habits, Primary health care, Relationship-centered care, Transpersonal caring

## Abstract

**Background:**

Previous studies have reported that organizational structures and cultures in primary health care are obstacles to district nurses doing successful work in health promotion practice (HPP). Because organizational structures are not easily changed, Jean Watson’s Attending Nurse Caring Model (ANCM) was introduced and piloted at a primary health care center in Sweden, aiming to transform HPP so as to empower district nurses and increase their work satisfaction.

**Aim:**

To investigate patients’ experiences of the caring encounter in HPP after introduction of the ANCM in Swedish primary health care, the aim being to explore the essential components of the caring encounter between patients and district nurses.

**Methods:**

A descriptive and qualitative research design was used. Data collection was performed using individual face-to-face interviews with twelve patients at risk for developing CVD. Data analysis consisted of both deductive content analysis, using a categorization matrix based on the ANCM and, subsequently, inductive latent content analysis.

**Results:**

The findings were abstracted into three themes: 1.‘Feeling the deepest essence of being cared for’: to be respected and being put at the center of the encounter; 2. ‘Feeling acceptance and worth’: being treated with openness and permissive attitudes, 3. ‘Being in a supportive atmosphere that promotes hope’: to feel trust and being trusted in the encounter, and being empowered by hope. The unifying main theme of the caring encounter was abstracted as ‘Experiencing human dignity’.

**Conclusion:**

The present study revealed that the essence of the caring encounter between patients and district nurses in HPP is to be unconditionally accepted in an environment that inspires hope and encouragement. The ANCM seems to be a promising model to use for strengthening the caring encounter and supporting CVD patients in making healthy lifestyle choices. However, further studies of qualitative and quantitative designs are needed to investigate what the ANCM can contribute to HPP in Swedish primary health care.

## Background

The World Health Organization (WHO) estimates that 80% of all cardiovascular diseases (CVDs) are related to lifestyle factors, including tobacco use, unhealthy diet, physical inactivity and excessive intake of alcohol [1]. According to Swedish legislation and regulations, the main responsibility for identifying individuals at risk for developing CVD lies within primary health care [2, 3]. District nurses play a key role in identifying these individuals and in promoting a healthier lifestyle among those at risk [4, 5]. Within their health promotion practice (HPP), they are equipped with effective tools such as motivational interviewing [6] and physical activity on prescription [7], which enable them to support individuals´ lifestyle changes [8, 9]. Despite being provided with these tools, working as a district nurse in primary health care can be quite challenging [10]. It has been reported that nurses who receive structural support at the workplace more often perceive their health promotion tasks as important and stimulating [11]. In contrast, nurses who lack that support report frustration and decreased motivation to perform HPP [11]. In line with these findings, we previously identified lack of organizational structure as one of the major obstacles to district nurses doing successful work with HPP among patients at risk for CVD [12]. In our previous study, we concluded that organizational structures and culture should be improved so as to support district nurses in their HPP [12]. Given that organizational structures are not easily changed, we recommended that HPP include more holistic elements of care, the goal being to empower district nurses and increase their work satisfaction [12]*.*

One way to transform HPP into a more holistic and sustainable practice for district nurses is to start with theories or models that emphasize caregivers’ own work-related health and well-being as a prerequisite for providing authentic care to patients. Following a literature review, Jean Watson’s Attending Nurse Caring Model (ANCM) was chosen as a suitable holistic caring model, because it puts clear focus on supporting the health and well-being of the caregiver as an important factor in strengthening relationship-centered care [13, 14]. The ANCM seeks to respect patients as whole human beings; it emphasizes that a human being cannot be separated from him−/herself, from others or the surrounding world [13, 14]. From the perspective of nurses, the ANCM offers a shared worldwide and professional culture that is grounded in relationship-centered caring, incorporating both existential and carative values [13, 14]. The ANCM consists of ten carative factors/processes (Table 1) that can assist in translating the theory into clinical practice [15].

**Table 1:** Carative factors and processes

Carative factors	Processes
1. Formation of humanistic system of values	Sustaining humanistic-altruistic values by practice of loving-kindness, compassion and equanimity with self/others.
2. Instillation of faith-hope	Being authentically present, enabling faith/hope/belief system; honoring subjective inner, life-world of self/others.
3. Cultivation of sensitivity to oneself and others	Being sensitive to self and others by cultivating own spiritual practices; beyond ego-self to transpersonal presence.
4. Development of a helping-trusting relationship	Developing and sustaining loving, trusting-caring relationships.
5. Promotion and acceptance of the expression of positive and negative feelings	Allowing for expression of positive and negative feelings – authentically listening to another person’s story.
6. Systematic use of creative problem-solving caring process.	Creatively problem-solving-‘solution-seeking’ through caring process; full use of self and artistry of caring-healing practices via use of all ways of knowing/being/doing/becoming.
7. Promotion of interpersonal teaching-learning	Engaging in transpersonal teaching and learning within context of caring relationship; staying within other’s frame of reference-shift toward coaching model for expanded health/wellness.
8. Provision for a supportive, protective and/or corrective mental, physical, sociocultural and spiritual environment	Creating a healing environment at all levels; subtle environment for energetic authentic caring presence.
9. Assistance with gratification of human needs	Reverentially assisting with basic needs as sacred acts, touching mindbodyspirit of spirit of other; sustaining human dignity.
10. Allowance for existential-phenomenological-spiritual forces	Opening to spiritual, mystery, unknowns-allowing for miracles.

Ref:© Watson Caring Science Institute 2016 https://www.watsoncaringscience.org/jean-bio/caring-science-theory/10-caritas-processes/

The model is further intended to support nurses in increasing their caring consciousness and their reflective activities by means of focus groups, meditation, or other experiential mind -body activities [13, 16]. The ANCM has primarily been applied within hospital settings in the US, where it was shown to promote health among health care staff and positive changes in relationship-centered patient care [13]. To the best of our knowledge, the ANCM has not yet been piloted in the context of primary health care, and not at all in Sweden.

As a first step to transform HPP and to include in it more holistic elements of care, we decided to introduce and pilot the ANCM among district nurses at a primary health care center in northern Sweden. The pilot study was developed based on the strategies found in the Medical Research Council guidelines [17] and conducted during a 5-month period in the spring of 2017. As a part of the pilot, five nurses participated in eight group sessions where they received training in the core concepts of the ANCM – how to apply the theory in clinical practice – and participated in experiential learning activities [18].

The present study was initiated to describe patients’ experiences of the caring encounter in HPP, after introduction of the ANCM at a Swedish primary health care center. A qualitative study design was chosen; the aim was to explore the essential components of the caring encounter with district nurses, as perceived by patients at risk of developing CVD. In the study, the caring encounter was regarded as a dialogue and/or relationship between two human beings who are dependent on each other in an intimate connection [19].

Obtaining in-depth insights into the caring encounter, from the perspective of patients, was regarded as an essential evaluation step in the process of transforming districts nurses’ HPP into a more holistic and sustainable practice. Results from an evaluation of district nurses’ personal experiences of and satisfaction with working with HPP after introduction of the ANCM will be published elsewhere.

## Methods

### Aim

To investigate patients’ experiences of the caring encounter in HPP after introduction of the ANCM in Swedish primary health care, the aim being to explore the essential components of the caring encounter between patients and district nurses.

### Design

To capture variations in patients’ experiences of the caring encounter, a descriptive qualitative research design was chosen using face-to-face interviews. Initially, a deductive [20] and, subsequently, an inductive latent content analysis inspired by Graneheim, Lindgren, and Lundman [21] were employed.

### Participants

Participants were recruited at a health care center in northern Sweden where the ANCM had been piloted. This health care center was not the first author’s (the interviewer’s) workplace, nor the work place of the other authors. To participate in the study, patients needed to be able to read and understand the Swedish language, have one or more risk factors for CVD, and have been engaged in one or several caring encounters in the HPP of district nurses who had participated in the ANCM pilot. Patients under the age of 18 or diagnosed with dementia were excluded.

An information letter with a request to carry out the study was sent by the first author to the management at the health care center in question. After verbal consent from the head manager, a list of 50 eligible patients was provided. These patients were then invited to participate in the study by mail through an invitation letter containing information about the objective of the study, stating that participation was voluntary and that they could withdraw from the study at any time, without any influence on their care. Written consent was hereafter obtained from each participant.

At study onset, both in the invitation/information letter and in connection with the interviews, each participant was informed about the researcher’s background, present occupation and about the study aims to explore the essential components of the caring encounter between patients and district nurses. Participants were not made aware of the researcher’s personal goals or further expectations. Since the ANCM pilot was solely targeted at district nurses, participants in this study were also not aware of the fact that district nurses at the healthcare center had participated in this pilot.

Out of the total of 50 patients who met the inclusion criteria, twelve gave their informed consent to participate in the study, 37 patients did not wish to participate, and one patient did not answer to the mail.

### Data collection

Individual face-to-face interviews were performed by the first author (KL) during the summer of 2017 at a neutral place nearby the health care center. The interviewer did not know the participants, i.e. no relationship existed beforehand. The interviews lasted between 40 and 90 min (mean 59 min, median 55 min). An interview guide was used to enable the variations in experiences of the caring encounter to be capture, and field notes were taken during the interviews. A broad open, explorative question was posed initially: ‘Could you please tell me about your encounter with your district nurse?’ Further questions were, for example, ‘In what ways were your needs met?’ ‘In what way did the district nurse give you support and instill confidence?’ ‘How do you feel the care you received will help you improve your health?’ ‘In what ways do you think you were respected when it comes to your own beliefs?’ The interviews were digitally audio-recorded and transcribed verbatim.

### Data analysis

The analysis was carried out by the first and last authors (KL and LPK). In the first step, the interview material was read through individually by the authors, to obtain an overall picture of the texts. The data analysis was initially deductive [20], based on the ten factors derived from the ANCM. A categorization matrix was developed that was then applied in a structural and systematic way to sort through, condense and analyze the transcribed interview text. The aim of using a matrix was to see the text through a filter that would allow identification of traces of the theoretical background of the ANCM when analyzing the patients´ descriptions of their experiences.

**Fig. 1:**
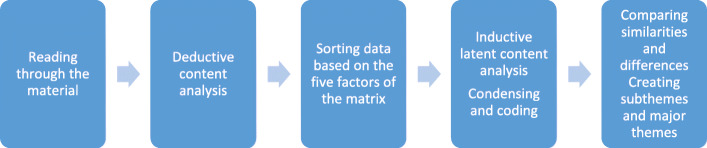
Analysis process

Initially, the categorization matrix included all ten factors. Data relevant to these factors were identified and sorted into appropriate factors. Subsequently, it was observed that several ANCM factors did not match the text material. Accordingly, our categorization matrix was revised and adjusted to five factors: *humanistic values, faith-hope, helping-trusting relationship, supportive protective environment and teaching-learning.* Subsequently, the matrix was used as a filter once more, and the text was sorted into factors matching the data content. In the next phase, the analysis work continued and transitioned into an inductive latent analysis, inspired by Graneheim et al. [21], the goal being to understand the material in more depth. In this phase, the data were condensed into meaning units, coded and sorted into similar groups. Based on similarities and differences, these groups were then abstracted into seven subthemes, which were finally summarized in three themes (Fig. 1, Table 2).

**Table 2:** Examples of the analysis process

Meaning units	Condensed meaning units	Subthemes	Themes
“…When I visit my nurse, I feel I am a human being, and she is also a human being, she is no God. And I dare to ask stupid questions and be unsure”	Being respected as a human makes me feel safe	To share human life	Feeling the deepest essence of being cared for
“… One is aware of it, and nobody wants to weigh 150 k. But to her a I can talk without feeling guilt. I even dare to tell her about the things I have failed to do”	Being overweight means being in a vulnerable situation. Experiencing an openness to talking about failures is good	To experience openness	Feeling acceptance and worth
”… Feeling a kind of support and good treatment, that is a source of security. Getting out of there and feeling hopeful”	Feeling support and being treated well give a feeling of hope	To be empowered by hope	Being in a supportive atmosphere that promotes hope

## Methodological considerations

To evaluate the trustworthiness of the present qualitative study, aspects of credibility, dependability, and transferability were addressed. In total, twelve agreed to be part of an interview, and face-to face individual interviews were then conducted. Having more than twelve interviews may have resulted in a richer variety in experiences. However, no further substantial variation was added during the final two interviews (Participant 11 and 12), leading to the judgment that the information power was sufficient and that a larger number of interviews would not have significantly altered the outcome of the thematic analysis [22].

Describing the research context and providing the participants´ demographics allow readers to make judgments concerning the relevance of the formulated themes and, thereby, the transferability of the findings. Credibility was established by presenting examples of the stepwise analysis process and interview excerpts. The first (KL) and the last (LPK) author were responsible for the analysis process; all authors agreed on the interpretation of the results. This collaboration in the research team ensured the dependability of the findings. Furthermore, the present paper was written according to the criteria for reporting qualitative research [23].

## Results

Table 3 describes the participants’ demographic characteristics. The participants consisted of seven males and five females with a mean age of 57 years. The majority had risk factors regarding CVD, as hypertension, physical inactivity and obesity. Three participants had diabetes type II.

**Table 3:** Demographics of participants

Number	Gender	Age	Marital status	* Risk factors for CVD	Occupation
*n* = 12	Male *n* = 7 Female *n* = 5	Median age 60 Mean age 57	Married/Living together *n* = 9 Single *n* = 3	Hypertension n = 9 Overweight *n* = 10 Obesitas *n* = 2 Diabetes type II n = 3 Tobacco use (Smoking) *n* = 0 Physical inactivity n = 10	Employed *n* = 8 Unemployed n = 2 Retired n = 2

* some participants had several risk factors

The findings of this study consisted of seven subthemes, three themes and finally one main theme that together described the variations in patients’ experiences of the caring encounter. The three themes were: (1) ‘Feeling the deepest essence of being cared for’ (2) ‘Feeling acceptance and worth’ and (3) ‘Being in a supportive atmosphere that promotes hope.’ The unifying main theme was named ‘Experiencing human dignity’ (Table 4). Each of these themes will be explored and exemplified in depth below. Interview excerpts are marked to indicate the participant making the statement: P1-P12.

**Table 4:** Factors, subthemes, themes and main theme

Factors	Subthemes	Themes	Main theme
*Humanistic values*	*To feel authenticity and genuineness*	*Feeling the deepest essence of being cared for*	*Experiencing human dignity*
*Teaching-learning*	*To share human life* *To be able to learn*		
*Faith-hope*	*To feel significant and to be respected* *To experience openness*	*Feeling acceptance and worth*	
*Helping-trusting relationship*	*To feel Trust – to be trusted*	*Being in a supportive atmosphere that promotes hope*	
*Supportive, protective environment*	*To be empowered by hope*		

### Feeling the deepest essence of being cared for

This theme consisted of three subthemes. Participants spoke about the importance of being listened to and respected as individuals. Some of them had earlier experiences of health conversations characterized by feelings of dissatisfaction and guilt, but in this context they spoke about encounters with district nurses whose actions were seen as genuine and authentic; they felt respected as human beings and like being put at the center of the encounter. This enabled participants to develop a good relationship with the district nurse.

#### To feel authenticity and genuineness

The participants talked about the feeling of being seen and paid attention to in a genuine manner. They regarded this essential in order to develop a good relationship with the nurse. Being met with real interest and respect for their perceptions and wishes, also seemed to increase the participants´ motivation to change their behavior concerning lifestyle habits.

*“She listens and I feel valuable, I am somebody and she believes in me” P10*

#### To share human life

The participants especially appreciated the familiar atmosphere in the caring encounters; this atmosphere seemed to help them feel confident and dare to talk about difficult things.

*“The staff are so skilled and have expert knowledge so I trust them, and they are being personal in a way, we share a lot of interests as well. I think it's the personal touch that matters*” *P6*

Participants perceived the nurse as non-judgmental and human, in the sense that participants felt comforted, were encouraged to share life events, and were reassured that no-one is perfect and that it is acceptable to make mistakes.

*“The way she treats me, puts her hand om my shoulder, says that everything is going to be all right even though I think I have failed, she is like a normal human being, if I say so, she understands me and seems genuinely interested in my life” P11*

#### To be able to learn

Receiving advice and support to the extent the participants were both interested in and in need of seemed to help them find answers and solutions for making changes they thought were best. This approach created a collaboration and constituted a relationship-enhancing ingredient.

*“She asks permission to explain the conditions to me and she always ends by asking if I have any further questions, which lets me reflect on my situation" P6*

### Feeling acceptance and worth

In this theme, two subthemes were formulated. Participants talked about previous feelings of failure when they, for example, had been unable to lose weight. They felt guilt and shame about coming back and not having succeeded. They compared these earlier experiences with the present one, describing how they now were being treated with openness and permissive attitudes.

#### To feel significant and to be respected

Participants with overweight felt vulnerable in the encounter with the nurse, specifically if they did not succeed in changing their lifestyle habits as agreed upon in previous encounters. Some spoke of earlier encounters as negative experiences, where they mostly felt they had failed and that they were less worthy than others. Participants were well aware of their failures, and did not want to be reminded of these negative encounters. Being treated in the present situation with acceptance, compassion and respect by someone who also had positive expectations, made them feel acknowledged as valuable individuals; they were growing as human beings and finding inner power and motivation.

*“She treated me with respect, I felt like everyone else, just as important as anyone else, if I can say that" P4*

Participants, who were treated in a respectful and understanding manner, felt that all kinds of questions that came to mind were allowed. Being able to learn without feeling inferior made a strong impression on them.

*“It was the nurse, how she spoke in a way, she made me understand how important it was to lose weight, there were no “pointers,” you know. She took time to explain things and I’m happy about it” P9*

#### To experience openness

Participants occasionally blamed themselves and took failures personally. Experiencing openness so that they were able to talk about these failures, was appreciated by the participants. To feel that the nurse was fully focused on one’s life, that the nurse attentively listened and was not distracted by anything else, was expressed as an overwhelming experience.

*“Nobody wants to weight 150 kilos. But to her, I can talk without feeling any guilt. I even dare to tell her about the things I’ve failed to do*, *I’ve never felt this before, and I’m so relieved” P4*

### Being in a supportive atmosphere that promotes hope

The final theme included two subthemes and described what had encouraged participants to believe in themselves as regards changing their lifestyle habits. Some of the participants had developed guilt and hatred toward themselves owing to external circumstances and poor self-esteem. Many of them had no one they could trust and had experienced little or no support from people in their surroundings, while simultaneously struggling with lifestyle habits.

#### To feel trust – to be trusted

Being able to meet on a deeper level was something that evolved. This led to insight, knowledge and mutual understanding, which were expressed in confidence and being believed in. The perception of being understood and feeling connected with another person seemed to both increase the participants’ feeling of hope and, ultimately, help them reconcile their problems.

*"It was the way she looked at me, she saw right into me, it was like she knew me, even though she didn't. At that point, I felt she trusted me, and I felt trust in her as well. I had hated myself for such a long time, even felt disgust for my body. I felt I was always wrong, said the most stupid things, blushed for the slightest thing, I hated myself. I had been overweight ever since I was little, I didn't know anything else, but I knew I was "wrong," even though I was a child. At school, I stopped raising my hand in class because I didn't want the other kids to look at me. I was called fatso and fatty and this hurt me a lot. Now that I’m grown up I was visiting my GP for something, and then it turned out that my blood sugar was a bit too high, not that I had diabetes but that I possibly could get it if I didn’t lose weight. So then I was invited to visit the nurse at the health care center for some dietary advice, it’s not that I don't know about this, I know it all, I’ve tried to lose weight all my life, but still … well, I came there and immediately I liked her. For the first time in a long time, I felt loved too" P10*

#### To be empowered by hope

Common to all of the participants, regardless of age and life situation, was that they were experiencing forgiveness as a force that helped them continue with life. Letting go of anger and hate was important to being able to push aside their guilt, shame and self-disparagement, enabling them to ask for forgiveness. Feeling the power of hope was a strengthening experience that engendered meaningful recovery. These conversations with the nurses had stimulated participants´ reflections on their own situation and behavior patterns and were described by a number of participants as helping them initiate or continue lifestyle changes.

*“It was necessary to forgive and move on. Only by forgiving I could start to like myself. This was the turning point. Suddenly I wasn't as bitter as before, I was able to observe myself from the outside. Understand that the people who were teasing me were only kids, as uncertain as I was. This made me finally feel I could make it. The nurse made me believe in myself and that I could manage to lose weight" P4*

### Experiencing human dignity

What best summarizes and describes the meaning of the participants’ experiences of the caring encounter is the feeling of experiencing human dignity (the main theme); this includes the assumption of sharing “being human,” which seemed to be the basis for the mutual caring encounters.

## Discussion

The present paper reports on one qualitative part of a larger pilot study aimed at investigating patients’ and nurses’ experiences of the caring encounter in HPP after introduction of the ANCM in Swedish primary health care. Overall, encouraging patients to describe their experiences resulted in three themes and one main theme, where the themes included a range of subthemes. These themes described a caring encounter in which one is unconditionally accepted in an environment that inspires hope and encouragement. Emerging from and unifying the sub- and major themes was the main theme, which was the feeling of experiencing human dignity.

Some of the participants’ experiences converged, and the views expressed were similar across all participants. For instance, the qualities of the nurse – such as selflessness, humility, trust and compassion – were perceived as having a significant effect on how the patient-nurse relationship unfolded. This is reinforced by Gustafsson et al. [24], who indicated that the occurrence of an encounter marked by caring and meaningful kindness is likely to depend on the nurse’s behavior and, moreover, seems to be decisive for how the encounter is perceived. Additional features distinguishing a caring encounter are common responsibility and sharing a nurturing fellowship, which ultimately provide life-changing moments and healing forces [24].

In the present study, positive, caring attitudes that fostered hope and comfort seemed both to support patients in making lifestyle changes and to empower them to move toward health-promoting behavior. Vatne and Nåden [25] stated that having a caring attitude and a willingness to communicate and be open are important ingredients in patients´ perception of the quality of the caring encounter – ingredients that help to further strengthen the relationship. Moreover, the present study demonstrated that openness and intimacy within the caring encounter are necessary conditions if patients are to believe in themselves. Being seen, heard and touched by another person – the nurse – meant sharing human life. Watson portrayed a genuine caring encounter as entering the life of another person and discovering the condition of that person’s spirit or soul [26]. Caring encounters of this kind support trust and promote feelings of acceptance; they are experienced as paving the way toward recovery and change of lifestyle habits. According to Barth and Näsholm [6], irrespective of the patient’s situation, encounters characterized by existential respectful treatment without judgment are effective in helping a person/patient make a decision to change and subsequently to initiate, implement and maintain that change.

To be respected, trusted, empowered and treated with openness and permissive attitudes were found among the essential components of the caring encounter in the present study. These findings are well in line with the outcome of a systematic meta-ethnographic analysis that aimed to understand how nurse–patient relationships can enhance patients’ health [27]. The six core themes that were identified in this study were: entering the patient’s world, trusting and telling, identifying different needs and uncovering change, patients becoming masters of their own health, patients experiencing health in illness, and nurses going the distance. The present study adds another essential component to this analysis, i.e. that of a supportive atmosphere that promotes hope. Feeling the power of hope was found to be a strengthening experience for patients, one that engendered meaningful recovery.

Some participants in the present study reported negative experiences of earlier health care encounters, particularly feelings of shame and guilt that had arisen in conversations about their weight. Some of the participants equated their obesity with moral failure; they believed their problems were self-inflicted and that they lacked willpower, overate, and had a sedentary lifestyle. These findings are in line with results presented by Mensinger, Tylka, and Calamari [28] and Ueland, Furnes, Dysvik, and Rørtveit [29]. Other participants talked about having met with a different nurse on every previous encounters, or about caregivers who were too busy to truly get involved, making it difficult to establish a relationship that enables trust and confidence. Relationship-based care must be built on continuity over time if an authentic connection is to be established [30].

### Strengths and limitations

This study has several strengths, including being among the first to pilot the ANCM in the context of primary health care and in Sweden. Furthermore, participants were not aware of the fact that the district nurses at the health care center had participated in the ANCM pilot. The results of the present study are therefore not influenced by possible expectations that the participants might have had in case they had known about introduction of the ANCM. Based on the present findings, it would seem reasonable to assume that the ANCM has to some extent contributed to the positive experiences of the caring encounters expressed by the participating patients. Nonetheless, it is one of the limitations of this study that the interviews were only conducted after the ANCM had been piloted, not prior to it, which might make it difficult to compare and conclude that patients’ positive experiences with their current caring encounter are directly related to the introduction of the ANCM. Another limitation to take into account is that some of the participants had met with their district nurse on several occasions during the study period. Therefore, the possibility that patients already had a well-functioning caring relationship with their district nurse cannot be excluded. Additionally, because continuity plays a role in building a trusting relationship, there may be a connection between the number of encounters patients had with their district nurse and their reported experiences. This was not specifically investigated.

### Implications for clinical practice and further research

The present findings are of value for HPP and the field of primary health care, as regards supporting patients at risk for CVD. It has been reported that Jean Watson’s ANCM can offer patients hope, healing and a belief in their inner ability [31], something that previously dissatisfied patients can benefit from when getting started with changing their lifestyle habits. Similar findings were reported by patients in the present study. When a patient with low self-esteem begins feeling accepted, significant to another person and respected for who he/she is, these feelings can help in building up self-esteem.

The potential of ANCM in supporting self-care and reflection among health care staff, is of interest for clinical practice. The guided introspection in the ANCM promotes self-reflection in nurses, and supports them in tending to their own health in a better way. This may also give nurses more insights in how their health status can positively or negatively affect a caring encounter [30]. Fostering and supporting self-care in future and present health care staff has been demonstrated to increase empathic characteristics [18, 32, 33], and is from a relational perspective of value when supporting patients and their inner resources. Improved empathy among health care providers is related to higher patient satisfaction, and better treatment outcome [34, 35].

Although the present study did not have a quantitative comparative design, it is reasonable to argue that the transpersonal caring theory underlying the ANCM influenced the district nurses’ approach in their caring encounters with patients. As reported by Koithan et al. [30], contributing to, and improving nurses communicative and empathic qualities support the development of trust in the caring encounter, which is regarded to be the foundation of relationship- and person-centered care.

This study is regarded as a first step towards evaluation of the ANCM in HPP in primary health care. The next step is to evaluate and publish on district nurses’ personal experiences of, and satisfaction with working with HPP after introduction of the ANCM in Swedish primary care. Since findings from the present study suggest that the ANCM may be a promising model for strengthening the caring encounter between patients and district nurses, further research is warranted. It is recommended to pilot the ANCM at more primary care health centers in Sweden in order to obtain a larger sample of patients and district nurses, and to quantitatively and qualitatively measure essential components of the caring encounter before and after the introduction of the ANCM, and in the longer term. These types of study designs should take into account the complex nature of whole system interventions [36, 37].

## Conclusions

The present study revealed that the essence of the caring encounter between patients and district nurses in HPP is to be unconditionally accepted in an environment that inspires hope and encouragement. The ANCM seems to be a promising model for strengthening this caring encounter and supporting CVD patients in making healthy lifestyle choices. However, further studies of qualitative and quantitative designs are needed to investigate what the ANCM can contribute to HPP in Swedish primary health care.

## Data Availability

The datasets generated and/or analysed during the current study are not publicly available because they contain confidential and sensitive data, but anonymously labeled data are available from the corresponding author on reasonable request.

## Abbreviations

CVD: Cardiovascular disease
ANCM: Attending Nurse Caring Model
HPP: Health promotion practice
